# Supplementary material for: Functional MICA Variants Are Differentially Associated with Immune-Mediated Inflammatory Diseases
Source: Int J Mol Sci. 2024 Mar 6;25(5):3036. doi: 10.3390/ijms25053036 (PMC10931785; doi:10.3390/ijms25053036)
Supplement: Supplementary file 1 [file ijms-25-03036-s001.zip › ijms-2851982-supplementary.pdf]

Supplemental Table S1. *MICA* non-synonymous cSNPs and major alleles identified in Taiwanese normal healthy controls and immune mediated diseases patients

Residue Position	SNP ID Number	Codon Changes	Exon	MICA Allele										
				001*	002	008	009	010	019	045	007	004	033	012
6R>P	rs9380254	CGT/CCT	Exon 2	G	-	-	-	C	-	-	-	-	-	-
14W>G	rs1063630	TGG/GGG	Exon 2	T	G	-	-	-	-	-	-	-	-	-
24A>T	rs1051785	GCT/ACT	Exon 2	A	G	G	G	G	G	G	G	G	G	-
36Y>C	rs1051786	TAT/TGT	Exon 2	G	-	A	A	A	A	-	-	A	A	-
122L>V	rs1051790	CTG/GTG	Exon 3	C	-	-	G	-	-	-	-	G	-	-
124T>S	rs41539919	ACT/TCT	Exon 3	A	-	-	-	-	-	-	-	-	T	-
**125E>K	**rs1051791**	GAG/AAG	Exon 3	A	G	G	G	G	G	G	G	G	G	G
129V>M	rs1051792	GTG/ATG	Exon 3	A	-	G	G	G	G	-	-	G	G	-
156H>L	rs3819268	CAC/CTC	Exon 3	A	-	-	-	-	-	-	-	-	-	T
173E>K	rs1051794	GAA/AAA	Exon 3	A	-	G	G	G	G	-	-	G	G	-
175G>S	rs1131896	GGC/AGC	Exon 3	G	-	-	A	A	A	-	-	A	A	-
181T>R	rs1131897	ACA/AGA	Exon 3	C	-	-	-	-	-	-	-	G	-	-
**206S>G**	rs1131898	AGC/GGC	Exon 4	G	-	A	A	A	A	A	-	A	A	-
**210R>W**	rs1051798	CGG/TGG	Exon 4	T	-	C	C	C	C	C	-	C	C	-
213I>T	rs1140700	ATA/ACA	Exon 4	C	-	T	C	T	T	T	-	-	-	-
**215S>T**	rs1051799	ACC/AGC	Exon 4	G	-	C	C	C	C	C	-	C	C	-
251Q>R	rs1063635	CAA/CGA	Exon 4	A	-	G	-	G	G	-	-	-	G	-

*MICA*001 allele was listed as the reference allele for the nucleotide sequence comparison with other MICA alleles that had allele frequency >1% in the combined population of Taiwanese normal controls and AS patients as shown in the Table 1.

**rs1051791 that causes amino acid (aa) substation at the residue 125 was not detected in Taiwanese but is listed to compare the nucleotide differences between MICA*001 and major MICA alleles identified in Taiwanese. Non-conservative residue changes are underlined. Residue substitutions that define two MICA lineages are in bold.
